# Factors associated with multidrug-resistant bacteria in healthcare-associated infections: a pediatric intensive care unit case-control study

**DOI:** 10.31744/einstein_journal/2022AO6704

**Published:** 2022-04-13

**Authors:** Barbara Barduchi Oliveira da Silva, Moacyr Silva, Fernando Gatti de Menezes, Eduardo Juan Troster

**Affiliations:** 1 Hospital Israelita Albert Einstein São Paulo SP Brazil Hospital Israelita Albert Einstein, São Paulo, SP, Brazil.

**Keywords:** Drug resistance, multiple, bacterial, Intensive care units, pediatric, Risk factors, Cross infection

## Abstract

**Objective:**

To investigate the prevalence of factors related to healthcare-associated infections, caused by multidrug-resistant bacteria, in a pediatric intensive care unit.

**Methods:**

A retrospective case-control study conducted from January 1, 2007 to December 31, 2018, in São Paulo (SP), Brazil. The study was carried out at the pediatric intensive care unit of a high-complexity, tertiary care general hospital. The study included patients aged 1 month to 19 years, admitted to the pediatric intensive care unit, diagnosed as healthcare-associated infections.

**Results:**

There was significant evidence of infection by multidrug-resistant bacteria associated with immunosuppressed patients (p<0.001), in whom the likelihood of multidrug-resistant bacteria infection was estimated to be nine-fold higher than among non-immunosuppressed patients (OR 8.97; 95%CI 2.69-29.94). In the analysis of multiple logistic regression model, we observed that immunosuppressed patients had an 8.5-fold higher chance of multidrug-resistant bacteria infection when compared to non-immunosuppressed patients (OR 8.48; 95%CI 2.54-28.35; p=0.001). There is evidence of association between the Case Group and presence of *Gram*-positive (p=0.007), coagulase-negative *Staphylococcus* (p<0.001), and *Gram*-negative (p=0.041) microorganisms.

**Conclusion:**

The immunocompromised-state variable is a factor related to healthcare-associated infections caused by multidrug-resistant bacteria, and the Case Group presented higher proportions of *Gram*-positive microorganisms and coagulase-negative *Staphylococcus*.

## INTRODUCTION

Healthcare-associated infections caused by multidrug-resistant (MDR) bacteria have increased,^(1)^and are considered a worldwide public health problem due to increased morbidity, mortality, and costs.^(2-5)^ In addition, healthcare-associated infections are the most frequent adverse event in children admitted to intensive care units (ICUs).^(6)^

Due to greater vulnerability of sick patients, use of invasive devices, and use of antimicrobials, MDR organisms and healthcare-associated infections are more frequently observed in ICU patients;^(1,2)^ therefore, ICUs are considered the epicenters of antibiotic resistance.^(7)^ Children are even more vulnerable, since their immune system is not fully developed and, during times of acute illness, both innate and adaptive immunities are compromised.^(2)^

Many studies have reported on incidences and factors associated with MDR bacteria in adult patients, but there are few studies available regarding the pediatric population.^(2,4)^ Knowledge of factors associated with MDR bacteria is considered important for the development of appropriate measures to control these infections.

## OBJECTIVE

To investigate the prevalence of factors related to health care-associated infections caused by multidrug-resistant bacteria, in a pediatric intensive care unit.

## METHODS

### Study design

We conducted a retrospective case-control study in the pediatric intensive care unit, of a reference high-complexity, tertiary care service, *Hospital Israelita Albert Einstein* (HIAE), in São Paulo (SP), Brazil, for private and health-insured patients, with 15 beds, mean census of 9.5 patients per day, during a study period of 12 years.

The study was conducted from January 1, 2007 to December 31, 2018. Data on patients admitted to the pediatric ICU and diagnosed with healthcare-associated infections during this period were retrieved from the hospital infection control service database.

The inclusion criteria were admission to pediatric ICU during the study period, age between 1 month to 19 years, and a confirmed healthcare-associated infections diagnosis. The cases of same-patient positive isolation were excluded, considering only the first event of infection per patient. Patients with fungal and/or viral infections were also excluded from the study.

The database contained 240 records of collections performed on pediatric patients, admitted to the pediatric ICU, presenting with healthcare-associated infections. After applying the exclusion criteria, 168 collections were identified. The Case Group was formed by all patients with an isolated agent resistant to at least one antibiotic, in three or more classes of drugs, with each one being considered the first collection in which this happened. Patients who did not present agents with antimicrobial resistance to were included in the Control Group, also considering the first collection for each patient. After excluding repetitions, the sample consisted of 38 patients in the Case Group and 59 in the Control Group.

To assess the association between the study group and age, patients were divided into the following categories according to the developmental stages of the pediatric patients: children under 1-year (infants), between 1 and 12 years (children), and over 12 years (adolescents).

To compare the distribution of microorganisms between the study groups, the isolated agents were grouped into classes, since some agents were not frequent. One occurrence per patient was considered.

- *Enterobacterales: Citrobacter freundii*, *Enterobacter spp*, *Escherichia coli*, *Haemophilusspp*, *Klebsiella pneumoniae*, *Morganella morganii ssp* and *Proteus spp*, *Serratia marcescens.*
- Non-fermenting *Gram*-negative *bacilli* (NFGNB): *Achromobacter spp*, *Acinetobacter spp*, *Bordetella bronchiseptica*, *Burkholderia cepacia*, *Pseudomonas aeruginosa* and *Stenotrophomonas maltophilia.*
- *Gram*-positive: *Enterococcus spp*, *Rothia mucilaginosa*, *Staphylococcus aureus*, coagulase-negative *Staphylococcus* and *Streptococcus spp.*

### Definitions

This study was conducted using the definition of healthcare-associated infections by the *Agência Nacional de Vigilância Sanitária* (ANVISA) and by the Center for Disease Control and Prevention (CDC), in which only those infections presented and identified more than 48 hours after admission to the health organization are considered healthcare-associated infections.^(8,9)^ Coagulase-negative *Staphylococcus* infection was defined as the presence of the agent in sterile liquid, in the case of blood cultures. In addition, coagulase-negative *Staphylococcus* infections were defined according to CDC criteria for each year of isolation.

Resistance to multiple drugs was defined as the non-susceptibility of one strain to at least one agent, in three or more classes of antibiotics.^(3,5,7)^

Cases were defined as those patients diagnosed with healthcare-associated infections due to MDR bacteria isolates. Cases were taken into consideration as of the first collection in which this occurred, and only the first occurrence of infection per patient was included. Controls were defined as those patients diagnosed with healthcare-associated infections due to non-MDR bacteria, also taking into consideration only the first occurrence of infection per patient.

Previous hospitalization was considered for those patients with a history of admission to any healthcare unit, during the previous 90 days, including home care and long-term care.

Antimicrobial use was considered if administration occurred during the 30 days before the collection of culture material.

Surgical procedures were considered if performed before collection.

Invasive devices considered were central venous catheter, enteral feeding tube, urine catheter, and/or invasive mechanical ventilation devices. These were considered if inserted at any time before culture collection. The urine catheter variable was considered present if it remained for 24 hours or more. Similarly, invasive mechanical ventilation was considered if the patient received invasive respiratory care for a period of, at least, 24 hours.

This study used the CDC definition of chronic diseases, that is, conditions that last for one year or more, and require continuous medical attention, and/or limit daily living activities.^(10)^

Immunosuppression status was defined as those patients whose immunological mechanisms were deficient due to immune disorders (*e.g*., HIV infection, congenital immunodeficiency disorders, and/or cancer), or immunosuppressive therapy (*e.g*., cytotoxic chemotherapy, anti-rejection medication -such as calcineurin inhibitors, nucleotide synthesis inhibitors, and use of and corticosteroids in doses for immunosuppression).^(11)^

### Microbiological methods

As *per* the organization policy, and according to the period of study, either the automated Vitek^®^ system (BioMérieux, Rio de Janeiro, Brazil) or the Maldi-Tof technique was used to identify bacteria. To identify antimicrobial sensitivity, the automated Vitek^®^ system and disk-diffusion were used and, when necessary, concentration gradient diffusion (Etest^®^, AB Biodisk, Solna, Sweden; (Liofilchem^®^, Roseto degli Abruzzi, TE, Italy). Susceptibility to antimicrobial agents was determined according to Clinical and Laboratory Standards Institute (CLSI) guidelines.

### Data source and statistical analysis

The analysis of antibiograms and the search for associated factors was done via digitized medical records. We collected information on all possible healthcare-associated infections risk factors, including clinical, demographic, and microbiological variables, from both clinical and laboratory records of hospitalized patients. Next, we compared cases and controls to evaluate the factors associated with the acquisition of MDR bacteria.

To compare the distributions of microorganisms among the study groups, the isolated agents were grouped into classes and one occurrence per patient was considered. To evaluate evidence of microorganism association by isolated classes between the study groups, the χ^2^ and Fisher’s exact test were used. To investigate an association of MDR bacterial infection with patient characteristics, the Mann-Whitney test was used.

Binary logistic regression, both simple and multiple, were used with the stepwise method of selecting variables in the multiple model. The results of the models were presented as estimated odds ratios (OR), 95% confidence intervals (95%CI) for odds ratios, and p-values with equality tests of odds-to-one ratios. The analyses were carried out with the aid of the SPSS program and the R and forecast packages were used to adjust the time series model. For all analyses, the significance level adopted was 5%.

### Ethical considerations

The study was approved by the *Hospital Israelita Albert Einstein* Ethics Committee, with waiver of informed consent, under the registration # 3391-18, # 2.889.616; CAAE: 95983918.2.0000.0071.

## RESULTS

### Clinical and epidemiological data

In the Control Group, the median age of patients (Table 1) was 1 year (first quartile: 0.4 years; third quartile: 7 years), and in the Case Group it was 4.5 years (first quartile: 1 year; and third quartile: 11 years). Classifying ages according to the stages of development in pediatric patients, there is no evidence of differences between groups (p=0.170) regarding the distribution of patients. There is also no evidence of differences between groups as to sex distribution of patients (p=0.273).

**Table 1:** Characteristics of pediatric patients according to the study group

Patient characteristics	Group		p value

	Control (n=59)	Case (n=38)	
Age, years			
Median (Q1-Q3)	1.0 (0.4-7.0)	4.5 (1.0-11.0)	
Minimum-maximum	0.0-16.0	0.2-18.0	
Age, years, n (%)			0.170*
<1	25 (42.4)	9 (23.7)	
1-12	26 (44.1)	22 (57.9)	
>12	8 (13.5)	7 (18.4)	
Sex, n (%)			0.273*
Female	30 (50.8)	15 (39.5)	
Male	29 (49.2)	23 (60.5)	

* χ^2^. Standard deviation.

Q1: first quartile; Q3: third quartile.

We observed significant evidence of association of MDR bacterial infection with immunosuppressed patients (p<0.001), where the chance of MDR bacterial infection was estimated to be nine-fold greater than among non-immunosuppressed patients (OR 8.97; 95%CI 2.69-29.94). There was no evidence of a significant association of MDR bacterial infection with the other patient clinical and epidemiological variables (p>0.05 in all models) (Table 2).

**Table 2:** Logistic models in simple approach results, evaluating the association between multidrug-resistant bacteria infection and clinical and epidemiological variables

Variables	Group		Logistic model	
	
	Control (n=59)	Case (n=38)	OR (95%CI)	p value
Previous hospitalization^*^ (n=49)	31 (63.3)	18 (36.7)	0.76 (0.33-1.76)	0.526
Chronic disease diagnosis (n=80)	47 (58.8)	33 (41.3)	1.69 (0.54-5.24)	0.367
Surgical procedures^†^ (n=70)	39 (55.7)	31 (44.3)	2.16 (0.80-5.79)	0.127
Central venous catheter^†^ (n=50)	26 (52.0)	24 (48.0)	2.04 (0.88-4.74)	0.095
Enteral feeding tube (n=74)	44 (59.5)	30 (40.5)	1.28 (0.48-3.39)	0.622
Urine catheter use (n=19)	12 (63.2)	7 (36.8)	0.88 (0.31-2.49)	0.816
Invasive mechanical ventilation^†^ (n=30)	17 (56.7)	13 (43.3)	1.25 (0.52-3.01)	0.613
Antimicrobial use (n=86)	50 (58.1)	36 (41.9)	3.24 (0.66-15.90)	0.148
Immunosuppressed status (n=19)	4 (21.1)	15 (78.9)	8.97 (2.69-29.94)	<0.001

Results expressed as n (%).

The reference categories have 1.00 in place of the odds ratio.

^*^ 56 controls and 37 cases; ^†^ 57 controls.

OR: odds ratio; 95%CI: 95% confidence interval.

We prepared a multiple logistic model, considering the variable response infection by MDR bacteria, and as explanatory variables those that presented p<0.20 in simple models. The following were included: surgical procedures, use of central venous catheters (CVC), use of antimicrobials, and immunosuppressed status.

Regarding variables included in the multiple model, we had incomplete data for two patients in the Control Group, who were automatically excluded. In the saturated model, we applied a stepwise process of variable selection, and the final adjusted model had only immunosuppression status as an explanatory variable.

We observed that immunosuppressed patients had an 8.5-fold greater chance of infection by MDR bacteria, when compared to non-immunosuppressed patients (OR 8.48; 95%CI 2.54-28.35; p=0.001).

Table 3 describes the types of infection by study group. There was no evidence of association between Case Group and type of infection (p=0.118).

**Table 3:** Types of infection of pediatric patients

Types of infection	Group		p value

	Control (n=59)	Case (n=38)	
Vascular access	2 (3.4)	1 (2.6)	
Asymptomatic bacteriuria	1 (1.7)	0	
Acute enterocolitis	2 (3.4)	0	
Laboratory-confirmed bloodstream	3 (5.1)	10 (26.3)	
Upper airways	1 (1.7)	0	
Intra-abdominal	1 (1.7)	0	
Surgical sites (organs/space)	8 (13.6)	1 (2.6)	
Deep, surgical site	2 (3.4)	0	
Superficial, surgical site	4 (6.8)	1 (2.6)	
Urinary tract	8 (13.6)	4 (10.5)	
Ear/eye/nose/throat	3 (5.1)	1 (2.6)	
Peritonitis	1 (1.7)	0	
Skin and soft tissue	4 (6.8)	3 (7.9)	
Respiratory (bronchial/tracheal)	11 (18.6)	12 (31.6)	
Respiratory (pneumonia)	8 (13.6)	4 (10.5)	
Central nervous system	0	1 (2.6)	

Results expressed as n (%). * Fisher’s exact test.

### Microbiological data

In the Case Group, 47 microorganisms were isolated. In the Control Group, 70 microorganisms were isolated. There is evidence of association between the Case Group and presence of *Gram*-positive (p=0.007), coagulase-negative *Staphylococcus* (p<0.001), and *Gram*-negative (p=0.041) microorganisms (Table 4). Comparing the groups, we observed that patients from the Case Group presented higher proportions of *Gram*-positive microorganisms (57.9% *versus* 30.5% in the Control Group) and coagulase-negative *Staphylococcus* (28.9% *versus* 3.4% in the Control Group), and a lower proportion of *Gram*-negative microorganisms (52.6% *versus* 72.9% in the Control Group).

**Table 4:** Microorganisms, by isolated class

Isolated agents	Group		p value

	Control (n=59)	Case (n=38)	
*Gram*-positive	18 (30.5)	22 (57.9)	0,007*
Coagulase-negative *Staphylococcus*	2 (3.4)	11 (28.9)	<0.001*
*Gram*-negative	43 (72.9)	20 (52.6)	0.041*
Enterobacteriales	24 (40.7)	14 (36.8)	0.706*
Non-fermenting *Gram*-negative	22 (37.3)	8 (21.1)	0.091*

Results expressed as n (%). * χ^2^.

## DISCUSSION

We observed that an immunosuppressed state is a factor related to healthcare-associated infections caused by MDR bacteria. However, no significant evidence with other clinical and epidemiological variables was observed. As to microbiological factors, we observed the Case Group presented a higher proportion of infections by *Gram*-positive microorganisms, specifically coagulase-negative *Staphylococcus.*

A retrospective case-control study conducted at an adult ICU showed the immunosuppressed state as an independent factor associated with resistance (p=0.001),^(12)^which does not differ from our findings in the pediatric population. Since the misuse of antimicrobials exerts direct selective pressure, by eliminating sensitive microorganisms and promoting the selection of resistant organisms,^(13)^ we speculate this result is due to the fact that immunosuppressed patients are more likely to acquire infection, and use antibiotics in greater quantities.

Studies corroborated the fact that age is not associated with the acquisition of MDR bacterial infections, as well as the present study.^(4,5,14,15)^However, there are studies that contradict our findings.

A retrospective observational study in children showed that the non-neonatal period was significantly associated with carbapenem-resistant *Acinetobacter baumannii* infection (p<0.05).^(16)^Another prospective observational study found that the rate of bacterial resistance, especially MDR *Escherichia coli* and extended-spectrum beta-lactamases (ESBL)-producing organisms, was higher in children under 1 year of age (p=0.026).^(2)^ Asensio et al. verified the risk factors for *Klebsiella pneumoniae* infection, and showed that an age of less than 12 weeks was the risk factor independently associated with MDR infection (OR infection 13.1).^(17)^ Nonetheless, the aforementioned studies may differ from our findings, because they are specifically for *Gram*-negative bacteria.

Likewise the present study, other investigations did not find an association of sex as a risk factor for MDR bacterial infection.^(2,12,14,15,18)^On the other hand, a prospective study in a single center showed that females were an independent risk factor for MDR hospital infections (p=0.02).^(4)^While one study observed that males were associated with a higher rate of MDR infection (p=0.21).^(19)^

The higher incidence of *Gram*-positive microorganisms does not corroborate previous literature. A multicenter, retrospective, cohort study with a nested case-control, found a higher incidence of *Gram*-negative bacteria (55%) in relation to *Gram*-positive bacteria (32%).^(6)^ El Mekes et al. demonstrated that 76% of bacterial infections in adult ICU patients were due to *Gram*-negative bacteria.^(7)^ However, other studies have identified coagulase-negative *Staphylococcus* as the most common pathogen related to bloodstream infections in pediatric ICUs.^(1,4)^According to McGrath et al., catheter-related bloodstream infections represent the most common nosocomial infection in pediatric patients.^(1)^Virano et al. verified that *Gram*-positive bacteria were prevalent among laboratory-confirmed bloodstream infections (68.4% of cases).^(20)^According to data by ANVISA, the microorganisms cited as the most prevalent etiological agents of primary bloodstream infections associated with central venous catheter, at pediatric ICU, in Brazil, were coagulase-negative S*taphylococcus*, with 549 cases,^(21)^ not differing from our findings.

Tfifha et al., did not demonstrate a relation between MDR organisms and antibiotic use,^(22)^ as seen in our findings. Nonetheless, most studies available in the literature associate MDR organisms and previous use of antimicrobials.^(4-6,16,23,24)^ As aforementioned, it is estimated that 40% to 80% of patients at pediatric ICU received antibiotics.^(2)^In light of this statistic, we speculated that due to the considerable rate of use of this class of drugs in this population, it may not have impacted on the difference in consumption between the study groups, and probably, with an increase in the sample size, this difference could be observed. Despite the non-evidence of association in our study, minimizing the unnecessary use of antibiotics is a fundamental principle of antimicrobial management.^(25)^

Due to its retrospective design, this study has some limitations including, bias of information, and the inability to control confounding variables (lack of information, such as length of hospital stay until the occurrence of healthcare-associated infections, and time of use of antimicrobials). Since the study was conducted at a single hospital, it is not representative of other centers. In addition, the pediatric ICU is composed of a highly heterogeneous population, characterized by different medical and surgical underlying diseases, which could influence the analysis and misrepresent the impact of different associated factors. Another limitation is the number of the sample that, when compared to other studies, is smaller. However, more than 300 studies carried out in the adult population have been published, while only 113 studies, in the past 12 years, have been carried out in pediatric patients.^(24)^To the best of our knowledge, this is one of the first pediatric studies on the prevalence of healthcare-associated infections caused by MDR bacteria conducted in Brazil, in addition to being a 12-year observational study.

The epidemiology of MDR bacteria varies according to geographic location and organization.^(5,21)^ Therefore, knowledge of MDR organism risk factors should be routinely sought after, as part of the decision-making-process regarding to antibiotic therapy.^(25)^Moreover, surveillance systems may be useful to understand the epidemiology of MDR bacteria in geographic regions, as well as from a global perspective.^(8)^

## CONCLUSION

The immunocompromised-state variable is a factor related to health care-associated infections, caused by multidrug-resistant bacteria, and the Case Group presented higher proportions of *Gram*-positive microorganisms and coagulase-negative *Staphylococcus.*
